# Solitary pancreatic lymph node metastasis from carcinoma of the breast: case report

**DOI:** 10.1186/1746-1596-5-29

**Published:** 2010-05-17

**Authors:** Giacomo Corrado, Giorgia Garganese, Gilda Fuoco, Arnaldo Carbone, Giovanni Scambia, Gabriella Ferrandina

**Affiliations:** 1 Gynecologic Oncology Unit, Department of Oncology, Catholic University of the Sacred Heart, L.go A. Gemelli, 1 - 86100, Campobasso - Italy; 2 Institute of Human Pathology, Catholic University of the Sacred Heart, L.go A. Gemelli, 1 - 86100 Campobasso, Italy

## Abstract

**Background:**

We report the first case of isolated pancreatic lymph node recurrence in a locally advanced breast cancer patient.

**Case:**

A 41-year old woman underwent radical mastectomy according to Madden and removal of axillary lymph nodes for multicentric infiltrating ductal carcinoma pathologically staged as pT2N2M0. After six years from primary diagnosis, and four years from the diagnosis of lung recurrence, she developed an isolated metastatic lesion to pancreatic lymph node. After surgical excision of metastasis, hormone therapy with Exemestane was begun. At 16 months of follow-up, the patient appears free of disease.

**Conclusion:**

Because metastatization to visceral organ carries a very unfavorable prognosis, we think that the clinical significance of the elevation of CA 15.3 serum levels in the early detection of recurrence and in monitoring metastatic disease during follow-up, should be not underestimated.

## Background

Breast cancer is one of the most common causes of cancer-related death in women [1]. Haematogenous metastases to lung, bone, liver and brain represent the most frequent sites of disease recurrence [2], and once they occur patients' clinical outcome is dismal.

Metastatic involvement of pancreas and its lymph nodes from another site is uncommon and accounts for approximately 2% of pancreatic malignancies [3]. In large autopsy series the prevalence of pancreatic metastases has been described to be as high as 6 to 11%, and renal cell carcinoma appears to be the most common primary tumor metastating to pancreas [4]

Metastatic involvement of pancreas from primary breast cancer is exceptional [2]. No reports have been previously published documenting metastatic involvement of pancreatic lymph nodes. We report the first case of isolated involvement of pancreatic lymph node in a patient with diagnosis of breast cancer.

## Case Presentation

A 41-year-old white woman presented in July 2003 with two nodules in the upper quadrants of the left breast and underwent radical mastectomy according to Madden and removal of 18 axillary lymph nodes for a multicentric infiltrating moderately differentiated ductal carcinoma (2 cm maximum diameter); lymphovascular space invasion was positive, and 8 lymph nodes were shown to contain malignant cells.

The tumor was positive for both estrogen receptors (ER) (90%) and progesterone receptors (PR) (70%), negative for human epidermal growth factor receptor 2 (c-erbB-2), and positive for Ki-67 (15%). The tumor was pathologically staged as pT2N2M0.

Postoperatively, the patient received 8 cycles of adjuvant chemotherapy with Epirubicin (4 cycles) and Cyclophosphamide, Methotrexate, Fluorouracil (CMF) protocol (4 cycles) plus radiotherapy (44 Gy) to the left hemithorax and left supraclavicular fossa. Treatment continued with Leuprolide in a monthly dose of 3.75 mg for two years, and Tamoxifen in a daily dose of 20 mg.

In March 2007, an increase in CA15-3 levels (54.9 U/mL; reference level, < 30) was documented in the absence of any sign of recurrence as assessed by imaging. Thoracic computed tomographic (CT) scan demonstrated a solitary small soft tissue mass of approximately 10 mm of diameter located in the middle lobe of the right lung attached to the mediastinal pleura. A total body CT-PET was positive.

She underwent surgical resection of the nodule and of the visceral pleura of upper lobe. Histology examinations showed the presence of metastatic breast carcinoma both in the middle lobe and visceral pleura of upper lobe. The tumor was positive for both ER and PR, and negative for c-erbB-2. Postoperatively, she received 6 cycles of chemotherapy with Docetaxel and hormone therapy with Letrozole.

**Figure 1:**
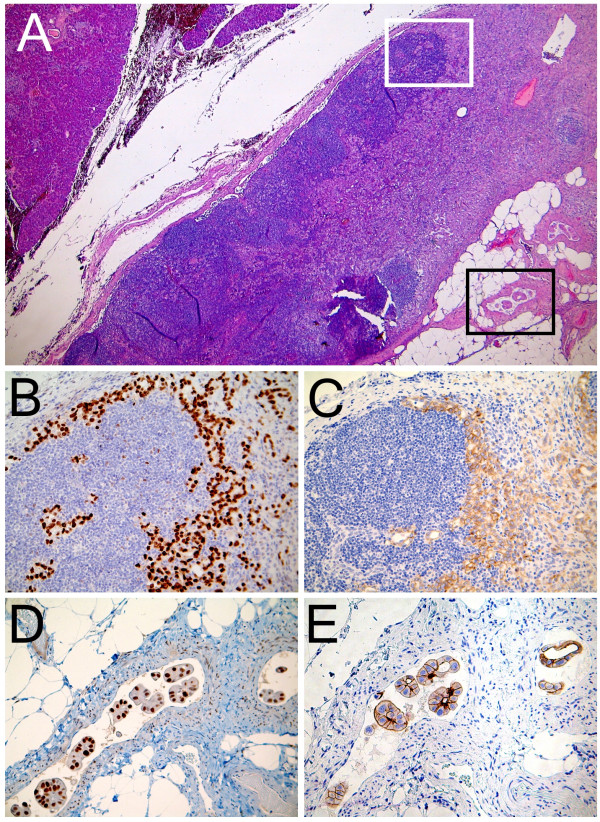
**Peripancreatic lymph node localization of breast carcinoma**. A) In this picture pancreatic tissue is observed in the upper-left corner. In the central portion the metastatic lymph node is observed. Lymph node measured 4×2 mm in section and presented heavily metastatic involvement. The area indicated by the white square is magnified in B and C. The area indicated by the black square is shown in D and E at higher magnification (hematoxylin and eosin, ×25). B) Virtually all neoplastic cells are strongly estrogen receptor positive (ER nuclear specific signal; LSABPx, ×200). C) Many tumor cells show a delicate membrane decoration for HER-2 (LSABPx, ×200). D and E) Neoplastic embolization is present, highlighted by staining for CK7 and 19 (data not shown). Note the strong nuclear positivity for ER (D) and the apparently stronger membrane signal for HER-2 (E), in these embolisms. Immunohistochemical characterization was made using a labeled streptavidin biotin peroxidase method (LSABPx). Visualization of the reaction was performed with the Dako LSAB 2 Kit peroxidase, containing labeled streptavidin biotin for primary rabbit/mouse antibody and diaminobenzidine (DAB).

Follow-up was negative until March 2009, when a novel increase of CA 15.3 (59.8 U/mL) was demonstrated. Ultrasonography of the liver showed, at the level of pancreatic head, a cystic lesion approximately 14 × 13 mm in diameter, with internal septa with features suggestive of intraductal papillary mucinous neoplasm. Pancreatic magnetic resonance confirmed this suspicion. Even if CT guided biopsy and a total body CT-PET were negative, the patient was strongly motivated to take away the pancreatic lesion, and a month later pancreaticoduodenectomy was performed; the postoperative course was uneventful. Pathological examination revealed a mucinous cystoadenoma of the pancreatic (1 cm maximum diameter) with negative resection margins. Surprisingly and fortunately, a peripancreatic lymph node removed during surgery (0.7 cm maximum diameter) was shown to contain tumour cells of the same histotype of primitive breast cancer and showing both ER and PR positivity, apparently stronger membrane signal for HER-2 (Figure 1), CK7 and 19 positivity (data not shown). Tumor cells resulted consistently negative for carcinoembryonic antigen (CEA) (data not shown) and for breast tissue marker gross cyctic disease fluid protein (GCDFP) (data not shown). Immunoreaction for CDX-2 antigen used to discriminate tumors of gastroenteric origin was negative (data not shown).

A re-joint evaluation of the slides was planned with two independent pathologists who confirmed the diagnosis.

Consequently, surgery tumour markers returned quickly within the reference range. After a careful counseling hormone therapy with Exemestane was begun which is currently ongoing. At 16 months of follow-up, the patient is free of disease.

## Conclusions

We describe a patient with breast cancer that after six years from primary diagnosis, and four years from the diagnosis of lung recurrence, she developed an isolated metastatic lesion to pancreatic lymph node. The peculiarity of this study is that, to our knowledge, this is the first case of isolated peripancreatic lymph node metastasis from breast cancer, and that the definitive diagnostic work up was decided only on the basis of the elevation of CA 15-3 level. In fact, all diagnostic examinations, including total body CT-PET scan and MRI of the pancreas, failed to show the real site of recurrence.

The usual sites of metastatic deposits from breast cancer include bone, liver, lymph nodes, lung and brain [5]. Pancreas is very rarely affected by metastasis from other primaries; in the largest series published to date (n = 973) of surgical specimens of pancreatic neoplasms [6] 31 (3.1%) were secondary tumors, of which were 11 lymphomas, 7 carcinomas from the stomach, 6 from the kidney, 2 from the lung, and 1 each from the liver, prostate, ovary, and uterus, as well as a case of Merkel cell carcinoma. In most cases, metastases developed after the primary tumor had been diagnosed, while in 34% of the cases they remained clinically silent and were documented only at autopsy as occult metastases [6]. Establishing the origin of neoplastic involvement of pancreatic lymph node was based on immunohistochemical results: indeed, in cancer of the breast cytokeratin (CK) 7 is required to be positive while CK-20 should be negative.

In our case there was a strong nuclear positivity for ER and an apparently stronger membrane signal for HER-2, in tumor embolisms. FISH analysis for HER-2, however, showed no gene amplification (LSABPx, ×200). Moreover, neoplastic embolization was present, highlighted by staining for CK7 and 19. Tumor cells resulted consistently negative for CDX-2. On the basis of this evidence, a relatively recent neoplastic colonization from breast cancer to the pancreatic lymph node could be hypothesized. As mentioned in the results, the suspicion of breast cancer recurrence was based only on the elevation of CA 15-3 serum level observed in the absence of any symptoms or imaging findings. According with the current guidelines published in February 2009 by the National Comprehensive Cancer Network (NCCN) [7], the recommended strategy for early detection of relapsed breast cancer involves history/physical examination, breast self-examination, mammography, and pelvic examination, while complete blood count, chemistry panel, bone scan, chest radiograph, liver ultrasound, CT scan, [18F]fluorodeoxyglucose PET scanning, magnetic resonance imaging, and tumor markers (CEA, CA15-3, TPA, etc) are not considered fundamental in the monitoring of breast cancer survivors.

However, several studies in the last decades have emphasized the role of the assessment of specific and appropriately used tumor markers as an easy, cheap, and significantly accurate tool for the early detection of distant metastases; recently, Nicolini et al. [8] showed that CA 15-3 as well as CEA, and TPA assessment contributes to an early diagnosis, which precedes by a few months, on average, clinical and/or instrumental signs of recurrent disease in 70% to 90% cases.

We think that the clinical significance of the elevation of CA 15.3 serum levels in the early detection of recurrence and in monitoring metastatic disease during follow-up, should be not underestimated, thus leading to plan a through instrumental diagnostic work-up and, in selected cases, an histologic evaluation.

## Consent

Written informed consent was obtained by patient for publication of this report and any accompanying images. A copy of the written consent is available for review by the Editor-in-Chief of this journal.

## Competing interests

The authors declare that they have no competing interests.

## Authors' contributions

All the authors contributed to the acquisition of data, revised the paper and gave final approval.
